# Is COVID-19 Keeping us Up at Night? Stress, Anxiety, and Sleep Among Adult Twins

**DOI:** 10.3389/fnins.2021.665777

**Published:** 2021-04-26

**Authors:** Siny Tsang, Ally R. Avery, Edmund Y. W. Seto, Glen E. Duncan

**Affiliations:** ^1^Department of Nutrition and Exercise Physiology, Washington State University Health Sciences Spokane, Spokane, WA, United States; ^2^Department of Environmental and Occupational Health Sciences, University of Washington, Seattle, WA, United States

**Keywords:** COVID-19, stress, anxiety, sleep problems, social restriction

## Abstract

In response to the COVID-19 pandemic, a variety of social distancing measures to mitigate the virus outbreak have been implemented. These measures may have unintended consequences on individuals’ well-being, such as increased stress, anxiety, and sleep disruptions. We investigated the extent to which individuals’ mental health status is associated with perceived changes in sleep amount and sleep quality among a sample of adult twin pairs (*N* = 909 pairs; 77% MZ, 23% DZ), less than a month after the outbreak was declared a pandemic by the World Health Organization. About half of participants reported no change in sleep amount (50.1%) or sleep quality (55.6%). Approximately one-third of the participants had increased amount of sleep (29.8%), and 32.9% reported a decrease in sleep quality. We found that stress and anxiety levels were associated with sleep reduction (ORs = 2.36 and 3.12 for stress and anxiety, respectively) and poorer sleep quality (ORs = 2.45 and 3.73 for stress and anxiety, respectively), even after taking into account between-family confounds. A much smaller association was observed between levels of stress and anxiety and increased sleep amount (ORs = 1.42 and 1.60 for stress and anxiety, respectively) and sleep quality (OR = 1.21 and 1.29 for stress and anxiety, respectively), which was no longer significant after controlling for between-family confounds. Our results demonstrate that stress and anxiety associated with the COVID-19 pandemic and social distancing measures may be linked to reduced sleep amount and quality.

## Introduction

The COVID-19 pandemic has upended the daily lives of people around the globe. A wide range of social distancing measures have been implemented, including limits on large gatherings, closing of schools, parks, and many public facilities, as well as lockdowns and/or shelter-in-place approaches. The stressful circumstances, combined with financial uncertainty and potential health risks, can have adverse impacts on individuals’ daily activities, as well as their psychological and physical health (Altena et al., 2020).

The impact of stress and anxiety on sleep has been well-documented (Van Reeth et al., 2000; Alvaro et al., 2013; Altena et al., 2016), such as disruptions in sleep after negative life events (Lavie, 2001; Gregory et al., 2006; Vahtera et al., 2007; Mezick et al., 2009), depression (Benca et al., 1992) or post-traumatic stress disorders (Ross et al., 1994; Lavie et al., 1998; Miller et al., 2017). As the COVID-19 outbreak became a pandemic, it is not surprising to observe sleep disruptions among individuals most impacted by the outbreak, such as patients or health care workers. For instance, high rates of sleep problems, such as difficulty falling asleep or waking up early, were found among a sample of medically isolated individuals in China (Xue et al., 2020). Healthcare workers (i.e., doctors, nurses, and health administrators) in China were more likely to report poor sleep quality than individuals in different occupational groups (Huang and Zhao, 2020). Among a sample of medical personnel treating COVID-19 patients, those with higher levels of acute stress and anxiety were more likely to have lower sleep quality (Xiao et al., 2020).

There has yet to be any published studies examining the extent to which stress and anxiety are linked to sleep problems in the US general population. Substantial heritability has been reported for sleep duration (Partinen et al., 1983; de Castro, 2002; Watson et al., 2006, 2010, 2014, 2017; McCall et al., 2019), sleep disturbance and pattern (Heath et al., 1990), and related components of sleep, such as electroencephalographic profile (De Gennaro et al., 2008), K-complex (Gorgoni et al., 2019). The objective of this study was to examine whether stress and anxiety levels were associated with perceived changes in sleep amount and sleep quality among a sample of adult twins, within the first month of social restriction measures implemented in response to the COVID-19 pandemic. The co-twin design is a type of natural experiment, offering a powerful alternative to random assignment, that can control for genetic and shared environmental confounds (McCall et al., 2019). Identical (i.e., monozygotic; MZ) twins share 100% of their genes, whereas fraternal (i.e., dizygotic; DZ) twins share 50% of their segregating genes, on average. Twin pairs raised in the same household are, by definition, matched for family and cultural background. By comparing twins in the family, genetically informed studies can adjust for a wide array of unmeasured individual characteristics which are similar within twins from the same household. We anticipated that individuals with higher stress and anxiety levels would be more likely to report a change, either decrease or increase, in sleep amount. We hypothesized a negative association between stress/anxiety with sleep quality, such that individuals with higher levels of stress and anxiety would be more likely to have decreased sleep quality, whereas those with lower levels of stress and anxiety would be more likely to report increased sleep quality.

## Materials and Methods

### Participants

Participants in this study were 909 same sex twin pairs (*N* = 1,818) from the Washington State Twin Registry (WSTR) who completed an online survey examining a number of health-related behaviors and outcomes and their impact due to COVID-19 mitigation. The WSTR is a community-based Registry of twin pairs, primarily recruited through Washington State Department of Licensing (DOL) records. Details regarding the recruitment procedures of the WSTR and additional information are reported elsewhere (Afari et al., 2006; Strachan et al., 2013; Duncan et al., 2019). Survey invitations were sent to 12,173 individuals registered and active in the WSTR between March 26 and April 5, 2020, approximately 1 week after the first a stay-at-home order became effective in California, US (The New York Times, 2020). Participation was voluntary and no incentive was offered. The study was approved by the Institutional Review Board at Washington State University. A wavier of documentation of consent was obtained, and consent was assumed by completing the questionnaire.

Response rates in the current study (32.6 and 21.2% individual and pair-wise response rate, respectively) were comparable to prior WSTR survey-based studies (∼32 and 21% individual and pair-wise response rate, respectively, across 13 unique studies). Demographic characteristics of the current respondents were relatively similar to those who completed a registry-wide study within the past year (Supplementary Table 1). A total of 3,971 individuals completed the survey, including 1,891 singletons (i.e., only one member of the twin pair completed the survey), 131 opposite-sex pairs (*n* = 262), and 909 same-sex pairs (*n* = 1,818). Only the 909 same-sex twin pairs (77% MZ, 23% DZ) were included in the twin analyses. Zygosity was determined using five questions in the WSTR enrollment survey asking about childhood similarity. Compared to biological zygosity indicators, the survey items correctly classify zygosity with at least 95% accuracy (Torgersen, 1979; Eisen et al., 1989).

### Measures

#### Perceived Stress

Perceived stress was assessed using the Perceived Stress Scale (PSS; Cohen et al., 1983). Participants responded to 10 items asking about their feelings and thoughts in the last 2 weeks on a 5-point Likert-type scale (0 = *Never*; 1 = *Almost never*; 2 = *Sometimes*; 3 = *Fairly often*; 4 = *Very often*). A total PSS sum score (range: 0–40) was computed by summing across all scale items, after reverse-coding responses to four positively stated items. Higher scores on the PSS reflect higher levels of perceived stress.

#### Anxiety

Anxiety was assessed using the anxiety subscale in the Brief Symptom Inventory (BSI; Derogatis and Melisaratos, 1983). Participants responded to six items asking how much discomfort each problem has caused them during the past two weeks including today on a 5-point Likert-type scale (0 = *Not at all*; 1 = *A little bit*; 2 = *Moderately*; 3 = *Quite a bit*; 4 = *Extremely*). A total anxiety score was computed by summing across all items (range: 0 – 24), where higher scores reflect higher anxiety levels.

#### Changes in Sleep

We asked participants a series of questions pertaining to changes in daily activities and behaviors using the prompt, “Compared to one month ago (i.e., prior to the spread of COVID-19), how much has your daily life changed in the following areas?” with five response categories: *decreased a lot, decreased somewhat, no change, increased somewhat, and increased a lot*. Participants’ responses to the two items assessing changes in sleep, “amount of sleep” and “sleep quality,” were used in the current study. Considering the atypical situation of the COVID-19 pandemic and differing societal responses across geographic location, we felt that participants’ perceived change in sleep could be a reasonable reflection of the actual changes (or lack thereof) in sleep.

As shown in Table 1, only very small proportions of the participants reported that their sleep amount decreased a lot (<4%) or increased a lot (<6%). Similarly, small proportions of the twins reported that their sleep quality decreased a lot (<6%) or increased a lot (<2%). These categories were combined with *decreased somewhat* and *increased somewhat*, respectively. All participants were subsequently categorized into *decreased*, *no change*, or *increased* for change in sleep amount and change in sleep quality.

**TABLE 1:** Descriptive statistics of select demographic characteristics, self-report change in sleep, perceived stress, and anxiety.

	Full sample (*N* = 3,971)	Same-sex twin pairs^*a*^ (*n* = 909 pairs)
Age	50.4 (16.0)	49.9 (16.0)
Gender		
Men	1,125 (30.8%)	444 (24.4%)
Women	2,746 (69.2%)	1,374 (75.6%)
Zygosity		
MZ	2,385 (60.1%)	1,400 (77.0%)
DZ	1,586 (39.9%)	418 (23.0%)
Change in sleep amount (%)		
Decreased a lot	111 (2.8%)	60 (3.3%)
Decreased somewhat	629 (15.9%)	291 (16.0%)
No change	2,042 (51.5%)	925 (50.9%)
Increased somewhat	978 (24.7%)	454 (25.0%)
Increased a lot	203 (5.1%)	87 (5.8%)
Change in sleep quality (%)		
Decreased a lot	197 (5.0%)	101 (5.6%)
Decreased somewhat	1,090 (27.6%)	495 (27.3%)
No change	2,194 (55.5%)	1,009 (55.7%)
Increased somewhat	412 (10.4%)	185 (10.2%)
Increased a lot	58 (1.5%)	21 (1.2%)
Perceived stress	12.3 (7.2)	12.6 (7.2)
Anxiety	3.6 (3.6)	3.8 (4.0)

*^a^Descriptive statistics are presented by individuals, not by twin pairs. Means (standard deviations) are presented for continuous variables. Frequencies (proportions) are presented for categorical variables.*

#### Covariates

Participants’ age and sex were included as covariates in the statistical analyses. Age referred to individuals’ age at which they completed the survey; it was computed based on the reported date of birth. Sex was self-reported as male or female.

### Statistical Analysis

**FIGURE 1:**
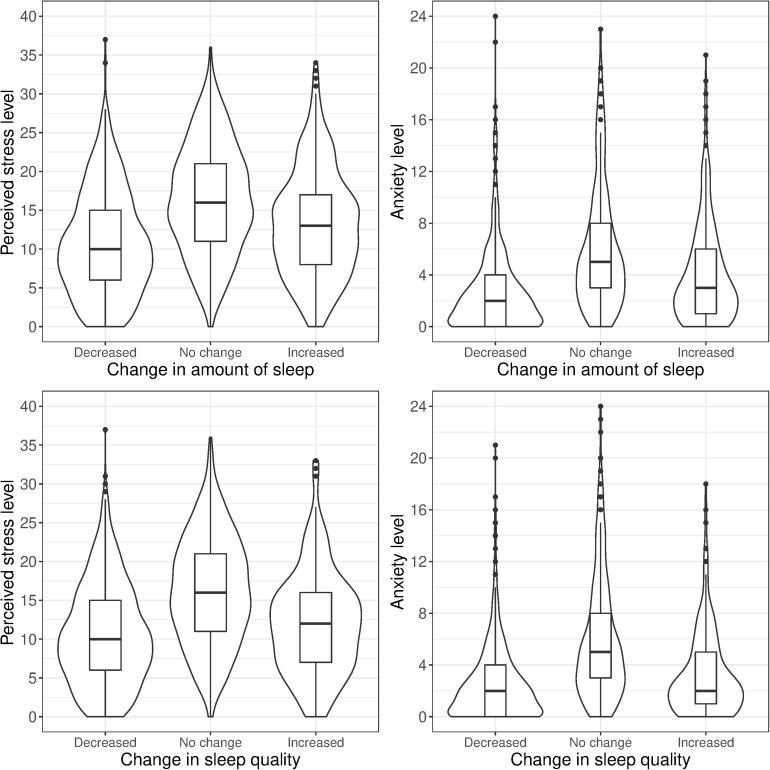
Stress and anxiety levels by self-reported change in sleep amount and sleep quality (same-sex twin pairs).

We carried out eight logistic regression comparisons: no change vs. decrease and no change vs. increase, each conducted separately for change in sleep amount and sleep quality, and separately on perceived stress and anxiety. Comparisons between no change vs. any change in sleep were performed separately because of the non-linear association between perceived stress and anxiety and the two changes in sleep outcomes (Figure 1).

**FIGURE 2:**
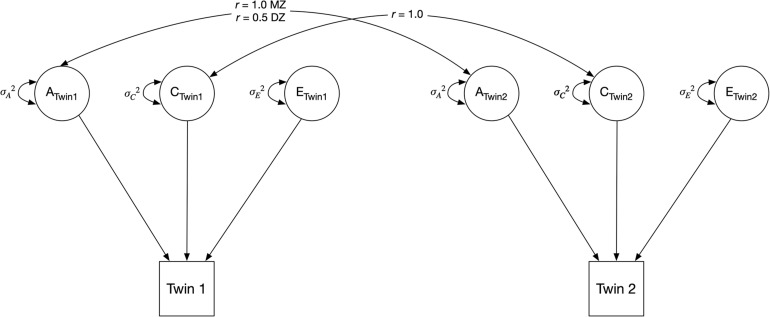
Classical univariate twin model. The variance of an observed variable is decomposed into three components, indicated by circles: additive genetic component (A), shared environmental component (C), and unique (i.e., non-shared) environmental component (E). MZ = monozygotic; DZ = dizygotic.

We began by fitting univariate twin models, partitioning the variances of each phenotype (stress, anxiety, and each change in sleep comparison) into additive genetic (A), shared environmental (C), and non-shared environmental (E) factors (Figure 2). The A variance components represent the additive effect of genes; it is correlated 1.0 between MZ twins (who share 100% of their genetic sequence) and 0.5 between DZ twins (who share 50% of their segregating genes, on average). The C variance components represent common (or shared) environmental experiences that make individuals raised in the same family similar to one another; they are correlated 1.0 for both MZ and DZ twins. The E variance components represent unique environmental experiences and include measurement error. They are uncorrelated between twins (Neale and Cardon, 2013). In the classical twin model, MZ (i.e., identical) twins share 100% of the additive effects of genes, therefore *r*_*A*_ = 1 (Figure 2). DZ (i.e., fraternal) twins share half (50%) of the additive effects of genes, thus *r*_*A*_ = 0.5. By definition, the shared environment is perfectly correlated for MZ and DZ twins raised together, therefore *r*_*C*_ = 1. The non-shared environment is not correlated for both MZ and DZ twins, so no correlated path is present between the E variance in Figure 2.

**FIGURE 3:**
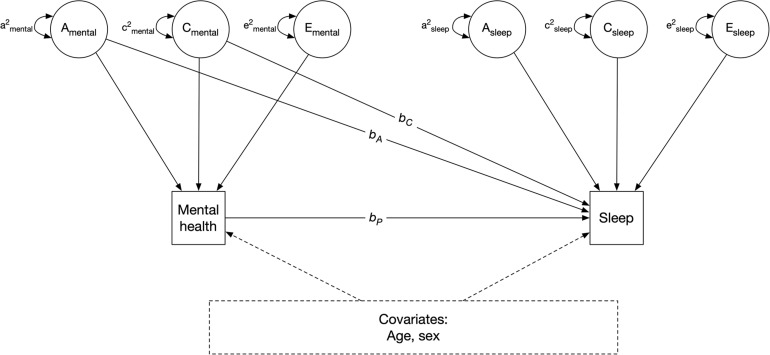
Quasi-causal twin model, controlling for age and sex. *A*: additive genetic component; *C*: shared environmental component; *E*: unique environmental component; *b*_*A*_ and *b*_*C*_: confounding genentic and shared environmental effect of mental health on sleep, respectively; *b*_*p*_: phenotypic association. Sleep refers to change in sleep amount or sleep quality, in separate models. Mental health refers to perceived stress or anxiety, in separate models.

Next, we fit simple phenotypic regression models to examine the association between stress or anxiety and change in sleep (Figure 3). The outcome of interest, change in sleep amount (or sleep quality, in separate models), was regressed on perceived stress (or anxiety, in separate models) to obtain an estimate of the observed relation between stress and change in sleep amount (*b*_*p*_ in Figure 3). This parameter (*b*_*p*_) represents the phenotypic association between perceived stress and change in sleep, without adjusting for the mediating effects of genetic and shared environmental confounds. It is equivalent to a population-level association.

We next re-estimated the models by simultaneously regressing change in sleep on phenotypic stress levels (*b*_*p*_), and on the shared additive genetic (A) and shared environmental (C) variance components of stress (*b*_*A*_ and *b*_*C*_) (Figure 3). *b*_*A*_ and *b*_*C*_ indicate the indirect effects of stress on change in sleep due to shared genetic and environmental components of stress, respectively. These are referred to as quasi-causal models; details and associated statistical methods are further described in Turkheimer and Harden (2014). The logic is that if identical (MZ) twins with different stress levels also differ in change in sleep, the association between stress and sleep change cannot be genetically mediated as MZ twins share 100% of their DNA. If MZ twins who differ in stress also differ in sleep change, this is consistent with the hypothesis that stress *causes* sleep change, at the phenotypic level. However, it is impossible to make definition inferences about causation without random assignment, such a relation is referred to as “quasi-causal.” Therefore, if the effect of perceived stress on change in sleep (*b*_*p*_) remained significantly different from zero after adjusting for between-family confounds (shared genetic and environmental components, *b*_*A*_ and *b*_*C*_, respectively), it would be interpreted as a *quasi-causal* effect. This means that, in a pair of MZ twins, the member of the pair with higher levels of stress is more likely to have change (or no change) in sleep than the co-twin with lower levels of stress. If, on the other hand, the effect of perceived stress on change in sleep (*b*_*p*_) was substantially reduced in magnitude and no longer statistically significant after controlling for the genetic and environmental (i.e., between-family) effects, a selection hypothesis would be supported. This means that a pair of MZ twins with different levels of stress would have similar likelihoods of reporting change (or no change) in sleep.

In the models examining the effect of perceived stress on change in sleep, the parameters *b*_*A*_ and *b*_*C*_ were initially estimated with large standard errors, indicating a high degree of correlation between the additive genetic (A) and shared environmental (C) variance of stress, and an insufficient power to differentiate between these two sources of covariation. To enable more stable estimates, *b*_*A*_ and *b*_*C*_ were constrained to equality in all subsequent models, meaning that the total between-family effect is estimated, rather than the individual additive genetic and shared environmental components. In the models examining the effect of anxiety on change in sleep, no *b*_*C*_ parameter was estimated as 0% of the variance in anxiety was attributable to shared environmental components. A final set of models were performed by including participants’ age and gender as covariates. Perceived stress and anxiety are both square root transformed as the two variables are positively skewed.

We provided descriptive statistics for both the full sample and the same-sex twins sample; twin analyses were performed only on the same-sex twins sample. Descriptive statistics were performed in the statistical program R 3.5.3 (R Core Team, 2015). All latent variable path analyses were conducted using the computer program Mplus v. 8.1 (Mutheén and Mutheén, 2012). The alpha level for testing hypotheses was initially set to 0.05. Considering that eight comparisons were performed, Bonferroni correction (Bland and Altman, 1995) was used; *p* = 0.006 (i.e., 0.05/8) was used as the significance level to adjust for multiple comparisons. Twin-based regression models are generally saturated; the only source of reduced fit involves incidental issues such as differences between twins arbitrarily assigned as Twin 1 and Twin 2 within pairs. All reported models fit the data closely using standard “goodness of fit” tests.

## Results

### Descriptive Statistics

Descriptive statistics for select demographic characteristics, perceived stress, anxiety, and the proportion of participants with different changes in perceived sleep amount and sleep quality for the full sample and same-sex twin pairs are shown in Table 1. Among the same-sex twin pairs sample, about half of the participants reported no change in sleep amount (50.1%), whereas smaller proportions reporting an increase (29.8%) or a decrease in sleep amount (19.3%). 55.7% of the twins reported no change in sleep quality, whereas smaller proportions reporting a decrease (32.9%) or an increase in sleep quality (11.4%). Perceived stress and anxiety levels are moderately correlated (*r* = 0.70, 95% CI = 0.67–0.72, *p* < 0.001); individuals who reported higher levels of perceived stress are also more likely to report having higher anxiety levels. Table 2 presents the cross-tabulation matrix of change in sleep amount and change in sleep quality [χ^2^(4) = 881.8, *p* < 0.001]. Less than half of the participants reported no change in both sleep amount and sleep quality (40%), with smaller proportions reporting similar changes in both sleep outcomes (16.1 and 10% for both decrease and both increase, respectively).

**TABLE 2:** Number and proportion of individuals with similar or different changes in sleep amount and sleep quality.

		Sleep amount		
		Decrease	No change	Increase
Sleep quality	Decrease	292 (16.1%)	175 (9.7%)	128 (7.1%)
	No change	53 (2.9%)	727 (40.2%)	229 (12.7%)
	Increase	5 (0.3%)	20 (1.1%)	181 (10%)

### Univariate Twin Models

Twin correlations and results of the univariate twin models of stress, anxiety, and change in sleep amount and quality are presented in Table 3. For perceived stress, most of the variance was attributable to the non-shared environmental component (61%), with smaller proportions attributable to additive genetics (23%) and shared environmental factors (16%). Fifty eight percent of the variance of anxiety was attributable to the non-shared environmental component, with the remaining proportion attributable to additive genetics factor (42%). There was substantial unique environmental variance in the two change in sleep amount comparisons (72 and 61% for increase and decrease vs. no change, respectively) and the change in sleep quality comparisons (89 and 77% for increase and decrease vs. no change, respectively). Model fit differences between saturated and nested models are presented in Supplementary Tables 2, 3.

**TABLE 3:** Twin correlations and standardized variance components for perceived stress, anxiety, and changes in sleep amount and sleep quality among same-sex twin pairs.

	*r*MZ	*r*DZ	*a*^2^	*c*^2^	*e*^2^
Perceived stress	**0.39 (0.03)**	**0.27 (0.06)**	0.23 (0.13)	0.16 (0.12)	**0.61 (0.03)**
Anxiety	**0.42 (0.03)**	**0.21 (0.02)**	**0.42 (0.03)**	–	**0.58 (0.03)**
Change in sleep amount					
No change vs. increase^a^	**0.28 (0.07)**	0.25 (0.06)	0.06 (0.41)	0.22 (0.39)	**0.72 (0.07)**
No change vs. decrease^a^	**0.39 (0.08)**	0.29 (0.20)	0.20 (0.43)	0.19 (0.40)	**0.61 (0.08)**
Change in sleep quality					
No change vs. increase^a^	0.11 (0.11)	0.06 (0.28)	0.10 (0.61)	0.01 (0.58)	**0.89 (0.11)**
No change vs. decrease^a^	**0.23 (0.06)**	**0.23 (0.07)**	–	**0.23 (0.09)**	**0.77 (0.06)**

*Standard errors are presented within parentheses. rMZ, monozygotic twin correlations; rDZ, dizygotic twin correlations. a^2^, c^2^, and e^2^: standardized biometric variance components obtained from classical twin model decomposing the variance of the phenotype into additive genetic, shared environment, and non-shared environment variance, respectively. ^a^Tetrachoric correlations are presented here due to the dichotomous nature of the comparisons. Bold values represent statistical significance at p < 0.05.*

### Perceived Stress and Sleep Amount

#### No Change vs. Decrease

As shown in Table 4, there was a positive phenotypic association between perceived stress and change in sleep amount (*b*_*p*_ = 0.869, OR = 2.36, *p* < 0.001). A one-unit increase in stress level was associated with a twofold increase in the likelihood of reporting a decrease in sleep amount. Although the strength of association was reduced after controlling for between-family confounds, the association remained statistically significant (*b*_*p*_ = 0.419, OR = 1.52, *p* < 0.001). Results were consistent after further controlling for age and sex (*b*_*p*_ = 0.423, OR = 1.53, *p* < 0.001).

**TABLE 4:** Unstandardized parameter estimates for phenotypic and biometric models estimating self-report change in sleep amount from perceived stress levels.

		No change vs. decrease			No change vs. increase		
		Est	OR [95% CI]	*P*	Est	OR [95% CI]	*p*
Phenotypic model							
	*b*_*p*_	0.869	2.36 [1.92, 2.90]	<0.001	0.354	1.42 [1.28, 1.59]	<0.001
Quasi-causal model^*a*^							
	*b*_*A*_	0.576	1.78 [1.45, 2.19]	<0.001	0.457	1.58 [1.32, 1.88]	<0.001
	*b*_*C*_	0.576	1.78 [1.45, 2.19]	<0.001	0.457	1.58 [1.32, 1.88]	<0.001
	*b*_*p*_	0.419	1.52 [1.34, 1.72]	<0.001	0.076	1.08 [0.98, 1.19]	0.127
Quasi-causal model^*a*^							
	*b*_*A*_	0.477	1.61 [1.17, 2.21]	0.003	0.423	1.53 [1.16, 2.01]	0.002
	*b*_*C*_	0.477	1.61 [1.17, 2.21]	0.003	0.423	1.53 [1.16, 2.01]	0.002
	*b*_*p*_	0.423	1.53 [1.35, 1.73]	<0.001	0.076	1.08 [0.98, 1.19]	0.132
	Age	−0.178	0.84 [0.79, 0.89]	0.009	−0.150	0.86 [0.82, 0.90]	<0.001
	Sex (F)	0.282	1.35 [1.07, 1.64]	0.009	0.019	1.02 [0.87, 1.20]	0.814
RMSEA [90%CI]		0 [0, 0.034]			0.029 [0, 0.048]		

*Phenotypic model does not include controls for between-pair confounds, whereas quasi-causal model include controls for between-pair confounds. Perceived stress is square root transformed; age is divided by 10. ^a^b_A_ and b_C_ are fixed to equal in the to allow for stable estimates. OR, odds ratio; b_A_ & b_C_: indirect effects of perceived stress on the change in sleep due to between-family effects (i.e., additive genetic and shared environmental components) of stress; b_p_: phenotypic association between predictor and outcome (full phenotypic effect in the phenotypic model, genetically informed phenotypic effect in the quasi-causal models); RMSEA: root mean square error of approximation.*

#### No Change vs. Increase

There was a phenotypic association between perceived stress and change in sleep amount (*b*_*p*_ = 0.354, OR = 1.42, *p* < 0.001; Table 4). Twins with higher stress levels were more likely to report an increase in sleep amount. This association was greatly reduced and became non-significant after controlling for between-family factors (*b*_*p*_ = 0.076, OR = 1.08, *p* = 0.127), as well as age and sex (*b*_*p*_ = 0.076, OR = 1.08, *p* = 0.132).

**FIGURE 4:**
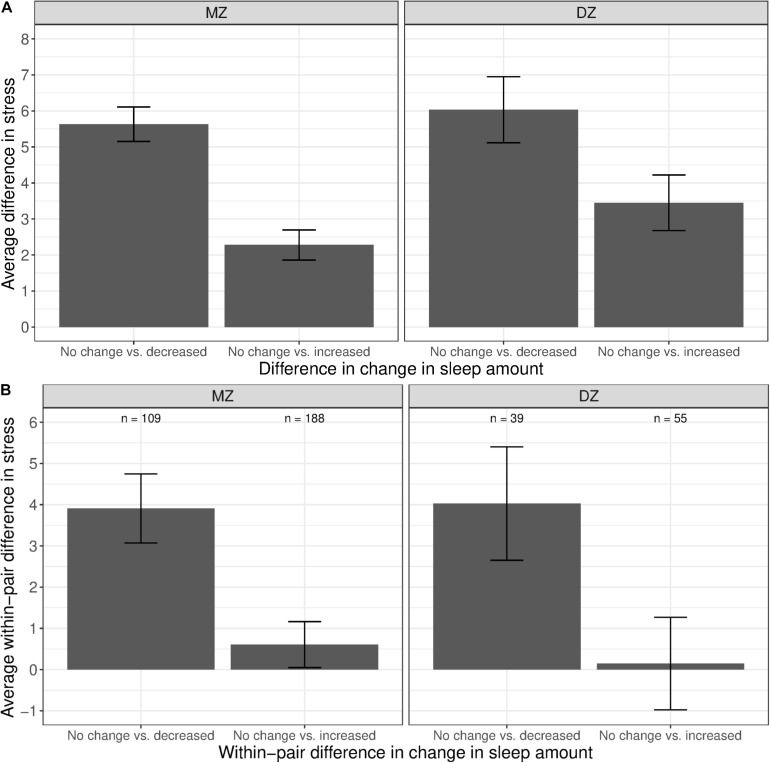
Average difference and within-pair difference in perceived stress levels between twin pairs with no change vs. change in sleep amount among same-sex MZ and DZ twin pairs. Error bars denote standard errors. **(A)** Average difference in perceived stress levels between twin pairs with no change vs. decreased/increased sleep amount. **(B)** Within-twin pair difference in perceived stress levels between twin pairs discordant in sleep amount.

Figure 4A shows the difference in average stress levels between participants who reported no change in sleep amount vs. those who reported a change in sleep amount, illustrating the phenotypic associations between stress and change in sleep amount. Twins with a decreased amount of sleep had substantially higher levels of stress than those with a similar amount of sleep (left bar in both panels). A much smaller association was observed when comparing between twins with an increased amount of sleep vs. those with no change in sleep amount (right bars in both panels). We computed the average within-pair difference in stress levels between discordant twin pairs (i.e., one member of the pair with change in sleep amount, and the other member of the pair with no change in sleep amount), separately for MZ and DZ twins. For each twin pair discordant in sleep change, a within-pair difference in stress levels was calculated by taking the difference between member of the pair with change in sleep amount and the co-twin with no change in sleep amount. The average within-pair difference in stress levels among twin pairs discordant in sleep change is illustrated in Figure 4B; pairs concordant in sleep (i.e., both members reported no change or both members reported a change) were not included. Among MZ twins where the between-pair confounds are controlled, stress levels were higher for the member of the pair with less sleep than their co-twin with no change in sleep amount (left bar in left panel). In contrast, there was minimal difference in stress levels between the member of the MZ pair with more sleep vs. their co-twin with no change in sleep amount (right bar in left panel), reflecting that the phenotypic association was confounded by between-family factors.

### Anxiety and Sleep Amount

#### No Change vs. Decrease

We found a phenotypic association between anxiety and change in sleep amount (*b*_*p*_ = 1.138, OR = 3.12, *p* < 0.001; Table 5). A one-unit increase in anxiety levels was associated with a threefold increase in the likelihood of reduced sleep amount. The association remained consistent after taking into account additive genetic confounds (*b*_*p*_ = 0.436, OR = 1.55, *p* < *0.001*) and demographic characteristics (*b*_*p*_ = 0.441, OR = 1.55, *p* < 0.001).

**TABLE 5:** Unstandardized parameter estimates for phenotypic and biometric models estimating self-report change in sleep amount from anxiety levels.

		No change vs. decrease			No change vs. increase		
		Est	OR [95% CI]	*p*	Est	OR [95% CI]	*p*
Phenotypic model							
	*b*_*p*_	1.138	3.12 [2.33, 4.17]	<0.001	0.472	1.60 [1.40, 1.84]	<0.001
Quasi-causal model							
	*b*_*A*_	0.640	1.90 [1.56, 2.31]	<0.001	0.373	1.45 [1.24, 1.70]	<0.001
	*b*_*p*_	0.436	1.55 [1.35, 1.77]	<0.001	0.146	1.57 [1.03, 1.30]	0.016
Quasi-causal model							
	*b*_*A*_	0.580	1.79 [1.36, 2.34]	<0.001	0.313	1.37 [1.09, 1.71]	0.006
	*b*_*p*_	0.441	1.55 [1.36, 1.77]	<0.001	0.153	1.17 [1.04, 1.31]	0.011
	Age	−0.181	0.83 [0.79, 0.88]	<0.001	−0.150	0.86 [0.82, 0.90]	<0.001
	Sex (F)	0.286	1.33 [1.08, 1.65]	0.009	0.018	1.02 [0.87, 1.19]	0.824
RMSEA [90%CI]		0.011 [0, 0.037]			0.021 [0, 0.043]		

*Phenotypic model does not include controls for between-pair confounds, whereas quasi-causal model include controls for between-pair confounds. Perceived stress is square root transformed; age is divided by 10. OR, odds ratio; b_A_: indirect effects of anxiety on the change in sleep due to additive genetic components of anxiety; b_p_: phenotypic association between predictor and outcome (full phenotypic effect in the phenotypic model, genetically informed phenotypic effect in the quasi-causal models); RMSEA: root mean square error of approximation.*

#### No Change vs. Increase

There was a significant phenotypic association between anxiety and change in sleep amount (*b*_*p*_ = 0.472, OR = 1.60, *p* < 0.001; Table 5). Twins who were more anxious were also more likely to report an increase in sleep amount. The association was no longer statistically significant after controlling for genetic factors (*b*_*p*_ = 0.146, OR = 1.57, *p* = 0.016), age and sex (*b_*p*_* = 0.153, OR = 1.17, *p* = 0.011).

**FIGURE 5:**
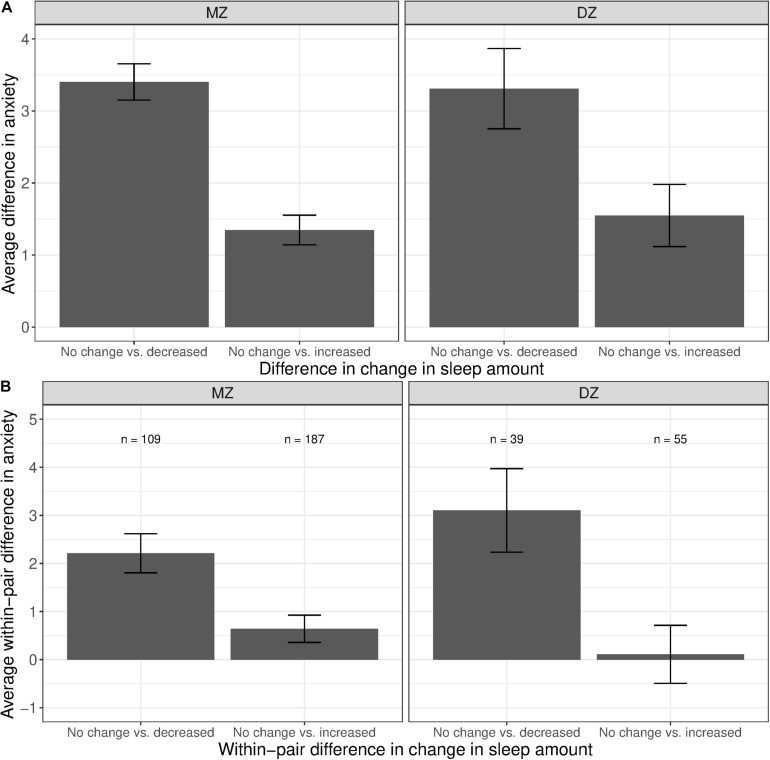
Average difference and within-pair difference in anxiety levels between twin pairs with no change vs. change in sleep amount among same-sex MZ and DZ twin pairs. Error bars denote standard errors. **(A)** Average difference in anxiety levels between twin pairs with no change vs. decreased/increased sleep amount. **(B)** Within-twin pair difference in anxiety levels between twin pairs discordant in sleep amount.

The phenotypic associations between anxiety and change in sleep amount are illustrated in Figure 5A. Compared with participants who reported no change in sleep amount, anxiety levels were higher among those with a decreased amount of sleep (left bars in both panels), as well as among those with an increased amount of sleep, though to a lesser extent (right bars in both panels). Figure 5B illustrates the average within-pair difference in anxiety levels between twins discordant in change of sleep amount; twin pairs concordant in sleep were not included. The average within-pair difference in anxiety levels is computed similarly as that in stress levels, described in the previous section. Higher levels of anxiety were observed among the member of the MZ pair who reported less sleep, as compared to their co-twin who reported no change in sleep amount (left bar in left panel). A difference in anxiety levels, though smaller in magnitude, was also observed between members of the MZ pair who reported an increased amount of sleep, when compared against their co-twin who reported no change in sleep amount (right bar in left panel).

### Perceived Stress and Sleep Quality

#### No Change vs. Decrease

There was a significant phenotypic association between stress and change in sleep quality (*b*_*p*_ = 0.898, OR = 2.45, *p* < 0.001; Table 6). Twins with higher stress levels were more likely to report a decrease than no change in sleep quality. This association was reduced, but remained significant, after controlling for between-family confounds (*b*_*p*_ = 0.430, OR = 1.54, *p* < 0.001), and further controlling for age and sex (*b*_*p*_ = 0.435, OR = 1.54, *p* < 0.001).

**TABLE 6:** Unstandardized parameter estimates for phenotypic and biometric models estimating self-report change in sleep quality from perceived stress levels.

		No change vs. decrease			No change vs. increase		
		Est	OR [95% CI]	*p*	Est	OR [95% CI]	*p*
Phenotypic model							
	*b*_*p*_	0.898	2.45 [2.03, 2.97]	<0.001	0.187	1.21 [1.07, 1.36]	0.002
Quasi-causal model^*a*^							
	*b*_*A*_	0.602	1.83 [1.55, 2.16]	<0.001	0.509	1.66 [1.30, 2.13]	<0.001
	*b*_*C*_	0.602	1.83 [1.55, 2.16]	<0.001	0.509	1.66 [1.30, 2.13]	<0.001
	*b*_*p*_	0.430	1.54 [1.38, 1.71]	<0.001	−0.115	0.89 [0.78, 1.02]	0.106
Quasi-causal model^*a*^							
	*b*_*A*_	0.544	1.72 [1.32, 2.24]	<0.001	0.588	1.80 [1.21, 2.67]	0.004
	*b*_*C*_	0.544	1.72 [1.32, 2.24]	<0.001	0.588	1.80 [1.21, 2.67]	0.004
	*b*_*p*_	0.435	1.54 [1.38, 1.72]	<0.001	−0.118	0.89 [0.77, 1.03]	0.106
	Age	−0.148	0.86 [0.82, 0.91]	<0.001	−0.123	0.88 [0.83, 0.94]	<0.001
	Sex (F)	0.499	1.65 [1.37, 1.98]	<0.001	−0.011	0.99 [0.81, 1.21]	0.917
RMSEA [90%CI]		0.011 [0, ‘0.038]			0.019 [0, 0.042]		

*Phenotypic model does not include controls for between-pair confounds, whereas quasi-causal model include controls for between-pair confounds. Perceived stress is square root transformed; age is divided by 10. ^a^b_A_ and b_C_ are fixed to equal in the to allow for stable estimates. OR, odds ratio; b_A_ and b_C_: indirect effects of perceived stress on the change in sleep due to between-family effects (i.e., additive genetic and shared environmental components) of stress; b_p_: phenotypic association between predictor and outcome (full phenotypic effect in the phenotypic model, genetically informed phenotypic effect in the quasi-causal models); RMSEA: root mean square error of approximation.*

#### No Change vs. Increase

We found a positive association between stress and change in sleep quality (*b*_*p*_ = 0.187, OR = 1.21, *p* = 0.002; Table 6), where twins with higher levels of stress were more likely to report an increase in sleep quality. This association was no longer significant after taking into account between-family factors (*b*_*p*_ = −0.115, OR = 0.89, *p* = 0.106) and further controlling for age and sex (*b*_*p*_ = −0.118, OR = 0.89, *p* = 0.106).

**FIGURE 6:**
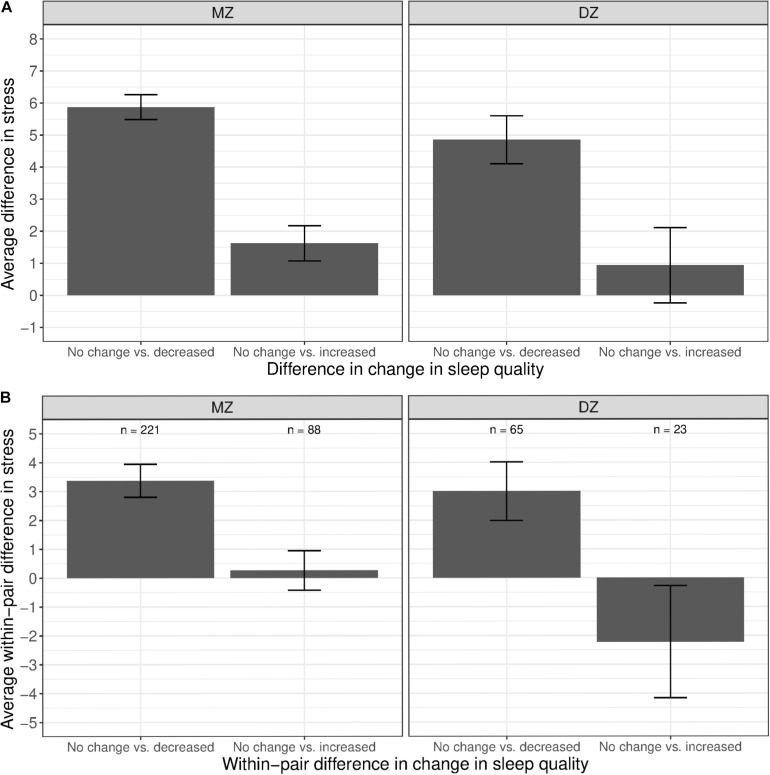
Average difference and within-pair difference in perceived stress levels between twin pairs with no change vs. change in sleep quality among same-sex MZ and DZ twin pairs. Error bars denote standard errors. **(A)** Average difference in perceived stress levels between twin pairs with no change vs. decreased/increased sleep quality. **(B)** Within-twin pair difference in perceived stress levels between twin pairs discordant in sleep quality.

As shown in Figure 6A, we observed a substantial difference in stress levels between twins with decreased sleep quality vs. those with no change in sleep quality (left bars in both panels). A similar difference, though to a lesser extent, was observed between MZ twins with an increased sleep quality vs. those with no change in sleep quality (right bar in left panel); the difference was further reduced and not different from zero among DZ twins (right bar in right panel). Within MZ twin pairs discordant in sleep quality change (Figure 6B), members of the pair with decreased sleep quality had, on average, higher stress levels than their co-twin with no change in sleep quality (left bar in left panel). However, there was no observable difference in stress levels between members of the pair with increased sleep quality and their co-twin with no change in sleep quality (right bar in left panel), illustrating that the phenotypic association was confounded by between-family influences.

### Anxiety and Sleep Quality

#### No Change vs. Decrease

We found a significant phenotypic association between anxiety level and change in sleep quality (*b*_*p*_ = 1.317, OR = 3.73, *p* < 0.001; Table 7). A one-unit increase in anxiety level was associated with an almost fourfold increase in the likelihood of decreased instead of no change in sleep quality. The association, though reduced in magnitude, remained significant after controlling for additive genetic confounds (*b*_*p*_ = 0.453, OR = 1.57, *p* < 0.001), and further controlling for demographic factors (*b*_*p*_ = 0.459, OR = 1.58, *p* < 0.001).

**TABLE 7:** Unstandardized parameter estimates for phenotypic and biometric models estimating self-report change in sleep quality from anxiety levels.

		No change vs. decrease			No change vs. increase		
		Est	OR [95% CI]	*p*	Est	OR [95% CI]	*p*
Phenotypic model							
	*b*_*p*_	1.317	3.73 [2.69, 5.18]	<0.001	0.255	1.29 [1.11, 1.49]	0.001
Quasi-causal model							
	*b*_*A*_	0.754	2.13 [1.81, 2.50]	<0.001	0.300	1.35 [1.12, 1.63]	0.002
	*b*_*p*_	0.453	1.57 [1.38, 1.79]	<0.001	0.002	1.00 [0.86, 1.17]	0.979
Quasi-causal model							
	*b*_*A*_	0.720	2.05 [1.64, 2.57]	<0.001	0.261	1.30 [0.99, 1.69]	0.056
	*b*_*p*_	0.459	1.58 [1.39, 1.80]	<0.001	0.009	1.01 [0.87, 1.17]	0.900
	Age	−0.152	0.86 [0.82, 0.90]	<0.001	−0.118	0.89 [0.84, 0.94]	<0.001
	Sex (F)	0.513	1.67 [1.38, 2.02]	<0.001	−0.008	0.99 [0.82, 1.20]	0.934
RMSEA [90%CI]		0.018 [0, 0.041]			0.023 [0, 0.044]		

*Phenotypic model does not include controls for between-pair confounds, whereas quasi-causal model include controls for between-pair confounds. Perceived stress is square root transformed; age is divided by 10. OR, odds ratio; b_A_: indirect effects of anxiety on the change in sleep due to additive genetic components of anxiety; b_p_: phenotypic association between predictor and outcome (full phenotypic effect in the phenotypic model, genetically informed phenotypic effect in the quasi-causal models); RMSEA: root mean square error of approximation.*

#### No Change vs. Increase

As shown in Table 7, anxiety level was associated with an increased likelihood of an increase in sleep quality (*b*_*p*_ = 0.255, OR = 1.29, *p* = 0.001). However, the association was small and reduced to non-significant after controlling for additive genetic components (*b*_*p*_ = 0.002, OR = 1.00, *p* = 0.979), and further control for age and sex (*b*_*p*_ = 0.009, OR = 1.01, *p* = 0.900).

**FIGURE 7:**
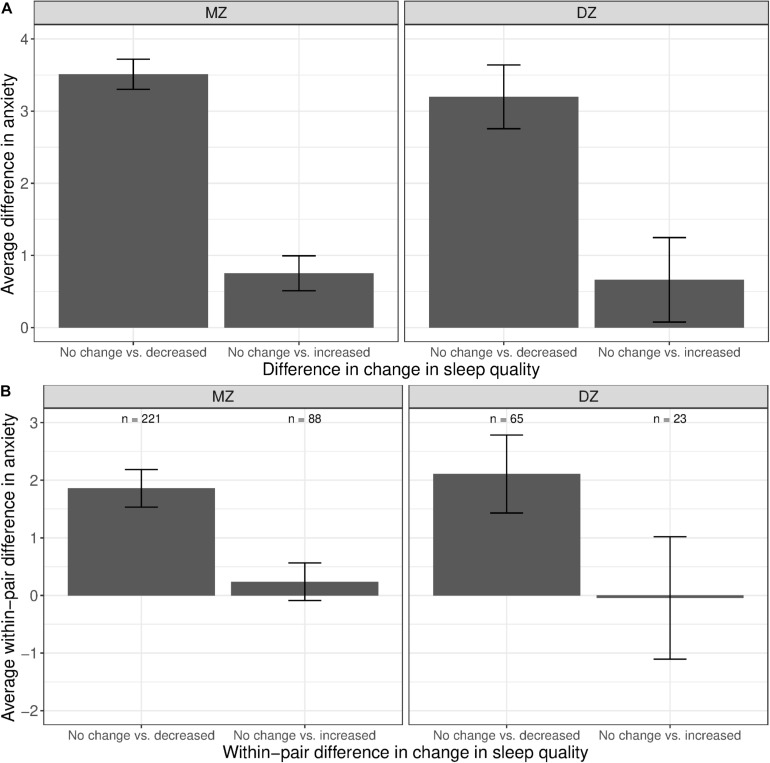
Average difference and within-pair difference in anxiety levels between twin pairs with no change vs. change in sleep quality among same-sex MZ and DZ twin pairs. Error bars denote standard errors. **(A)** Average difference in anxiety levels between twin pairs with no change vs. decreased/increased sleep quality. **(B)** Within-twin pair difference in anxiety levels between twin pairs discordant in sleep quality.

The phenotypic associations between anxiety and change in sleep quality are shown in Figure 7A. Twins with increased sleep quality had, on average, higher anxiety levels than those who reported no change in sleep quality (left bars in both panels). Although twins with increased sleep quality also had higher mean anxiety levels than those with no change in sleep quality, the difference was much smaller (right bars in both panel). Within MZ twin pairs discordant for change in sleep quality, the member of the pair with decreased sleep quality had higher anxiety levels than their co-twin with no change in sleep quality (Figure 7B, left bar in left panel). In contrast, there was no visible difference in the mean levels of anxiety between the member of the MZ pair with increased sleep quality vs. their co-twin with no change in sleep quality (right bar in left panel).

## Discussion

Our results demonstrate that individuals with higher levels of stress and anxiety were more likely to report a decrease, rather than no change, in sleep amount and sleep quality. These phenotypic associations remained robust when controlling for between-family confounds. Within MZ twin pairs, the member of the pair with higher stress or anxiety levels was more likely to sleep less and with poorer sleep quality compared to their co-twin with lower stress or anxiety levels. These findings suggest that stress and anxiety feelings during the COVID-19 pandemic may be linked to reduced amount and quality of sleep.

We found similar associations between mental health and increased sleep amount and sleep quality. Individuals with higher levels of stress and anxiety were more likely to report an increase, rather than no change, in sleep amount and sleep quality. After controlling for between-family confounds, only the association between anxiety and increased sleep amount remained robust. Within MZ twins, the member with higher anxiety levels was more likely to report more sleep than their co-twin with lower anxiety levels. However, this association was not statistically significant after adjusting for multiple comparisons. In contrast, the associations between stress on increased sleep amount and sleep quality, and that between anxiety on increased sleep quality, were likely due to confounding by between-family factors. After controlling for between-family confounds, the average within-pair difference in stress levels was small and highly variable between those with more or no change in sleep amount. Similarly, there was minimum within-pair difference in stress and anxiety levels between the member of the MZ pair with increased sleep quality vs. their co-twin with no change in sleep quality.

Since the relationships between mental status and change in sleep amount and sleep quality was examined separately in the current study, we explored whether similar associations would be observed when comparing individuals who reported consistent changes in sleep amount and sleep quality (i.e., decrease, no change, or increase in both sleep amount sleep quality). Preliminary results were consistent with findings in the current study; perceived stress and anxiety levels were substantially higher among those who reported a decrease in both sleep amount and sleep quality (*n* = 292, *M* = 16.5, *SE* = 0.40 for stress; M = 6.3, *SE* = 0.29 for anxiety) than those who reported no change in both sleep amount and sleep quality (*n* = 727, *M* = 9.6, *SE* = 0.23 for stress; *M* = 6.3, *SE* = 0.29 for anxiety). Perceived stress and anxiety levels were also higher among those who reported an increase in both sleep amount and sleep quality (*n* = 181, *M* = 11.8, *SE* = 0.49 for stress; *M* = 3.1, *SE* = 0.23 for anxiety) than those who reported no change, though to a much lesser extent. Although we were unable to examine whether these phenotypic associations remained robust after controlling for between-family confounds due to limited sample size, our findings suggested that individuals’ mental health status may have a similar relation with the two components of sleep.

Findings in our study provide preliminary evidence that the psychological burden of the COVID-19 pandemic may be associated with reduced sleep amount and sleep quality. These results add to the growing literature illustrating that the COVID-19 pandemic may be linked to sleep problems (Pérez-González et al., 2020). It has been reported that over half of the participants in an Italian adult sample reported impaired sleep quality and sleep habit (Franceschini et al., 2020). Among a sample of adults in Greece, 37.6% of the participants had sleep problems during this pandemic (Voitsidis et al., 2020). Compared to men, women were more likely to suffer from sleep problems (Guadagni et al., 2020), a finding also observed in our sample of adult twins, though the association was very small and only present in the no change vs. decrease comparisons (ORs range = 1.33–1.67). A study surveying a sample of Chinese residents in Wuhan and surrounding cities showed that individuals who endorsed more post-traumatic stress symptoms (PTSS) were more likely to have poorer sleep quality (Liu et al., 2020). Studies have also found associations between the pandemic and dream quality and quantity; individuals reported more negative emotions in dreams (Schredl and Bulkeley, 2020; Gorgoni et al., 2021), increased pandemic-related dreams (MacKay and DeCicco, 2020; Pesonen et al., 2020). Among frontline medical workers, reduced sleep duration and efficiency were linked to frequent nightmares during the COVID-19 pandemic (Lin et al., 2021), highlighting the possibility of burnout and/or development of PTSD symptoms. Taken together with existing epidemiolocal studies showing the relation between sleep disturbances and negative health outcomes, such as obesity (Buxton and Marcelli, 2010), impaired metabolic health (Schmid et al., 2015), type 2 diabetes (Knutson et al., 2006; Lee et al., 2017), and PTSS (McCall et al., 2019; Richards et al., 2019), the current pandemic may have brought about additional adverse psychological and health consequences, beyond those directly linked to the virus outbreak itself.

### Strengths and Limitations

The timing of the data collection was one of the major strengths of the current study, as our survey was administered less than a month after the World Health Organization (WHO) declared the novel coronavirus outbreak a pandemic on March 11, 2020 (World Health Organization [WHO], 2020). At the time of data collection (last week of March and first week of April in 2020), every state in the US had made an emergency declaration, in the form of a State of Emergency or Public health Emergency (Kaiser Family Foundation, 2020). Because we did not measure individuals’ mental health and daily behaviors immediately prior to the pandemic, we were unable to investigate whether respondents’ stress and anxiety levels, or actual amount and quality of sleep, had changed in response to the virus outbreak. Nonetheless, individuals’ perceived change in sleep amount and sleep quality provided us preliminary evidence that sleep has been disrupted during this time period, as individuals adjust to the uncertainty of the virus outbreak and implementations of social restriction measures.

Our genetically informed study allowed us to tease out the extent to which associations between mental health status and change in sleep amount and quality were due to between-family confounds. We showed that much of the association between perceived stress and anxiety and increase in sleep amount and sleep quality was attributable to between-family confounds. In contrast, the relation between mental health status and a decrease in sleep amount and sleep quality was robust, after taking into account between-family confounds.

Despite these strengths, the current study may be affected by self-selection bias. Although the response rates for this study were comparable to other survey studies conducted through the WSTR (∼30 and 20% individual and pair-wise, respectively), it is possible that these individuals’ responses to questions regarding stress and anxiety levels or changes in sleep amount and quality may not be generalizable to all individuals in the registry, nor to other population samples with different demographic characteristics. Our analyses were limited due to the current sample size, especially the small number of participating DZ men—we did not have enough statistical power to tease out the additive genetic and shared environment influences, and/or to perform a multiple group analysis for a stringent test of gender effect.

We relied on self-reported data in the current study. It is possible the questions on stress and anxiety levels addressed participants’ general mental health status, rather than acute feelings specific to the pandemic and/or corresponding social restriction measures. Changes in sleep amount and sleep quality were also self-reported; it is unclear the extent to which individuals’ perceived changes in these sleep components reflect actual changes in sleep amount and quality. It is possible that participants’ recollections of the changes in sleep may have been influenced by the pandemic and/or other confounding measures. Furthermore, data in this study is cross-sectional. As mental health status and sleep components were not assessed prior to the COVID-19 pandemic, it is unclear whether participants’ stress and anxiety levels have changed since the outbreak. We are also unable to ascertain whether the associations between mental health status and sleep disruptions were due to the coronavirus outbreak, social restriction strategies, and/or other factors that were not measured in our study.

Nonetheless, the current results provide the basis for on-going longitudinal studies examining the associations between mental health status and sleep disruptions. As society eases social restriction measures, follow-up studies could provide insights into how mental health status and sleep patterns change over time, the extent to which these variations correspond to changes in the outbreak development and/or corresponding societal responses, and whether one is more likely to affect the other (e.g., stress levels influence sleep amount or sleep amount affects stress levels). Future studies can also make use of smart electronic devices (e.g., exercise trackers) to assess individuals’ sleep amount and sleep quality in real time, providing a more accurate assessment of different sleep components. Relatedly, it would be worthwhile to explore the extent to which individuals’ perceived changes in sleep reflect actual changes in their sleep amount and sleep quality.

## Conclusion

In conclusion, our study found negative associations between mental health and a decrease in sleep amount and quality. Even after controlling for between-family confounds, individuals with higher levels of perceived stress and anxiety were more likely to have disruptions in sleep, with less sleep and poorer quality of sleep. On the other hand, individuals with higher stress and anxiety levels were also more likely to sleep more and had better sleep quality, though the association was smaller and no longer significant once taking into account between-family confounds. The current study showed that the stress and anxiety may be linked to sleep disruptions, particularly reduced amount of sleep and poorer sleep quality, during the COVID-19 pandemic. It is possible that the COVID-19 pandemic and corresponding social restriction measures may have unintended negative consequences for the general public—stressors related to the virus outbreak, financial burden, and changes in daily activities due to societal restrictions. It is important to continue monitoring the changes in daily behaviors and psychological consequences of COVID-19 in order to establish intervention strategies to help the public adjust to societal changes as a result of the pandemic and prepare for future outbreaks of similar scale.

## Data Availability Statement

The datasets presented in this article are not readily available because data is provided by the Washington State Twin Registry after acceptance of a manuscript proposal with a signed data access agreement. Requests to access the datasets should be directed to www.wstwinregistry.org.

## Ethics Statement

The studies involving human participants were reviewed and approved by the Washington State University Institutional Review Board. The ethics committee waived the requirement of written informed consent for participation.

## Author Contributions

ST contributed to the main analysis of the results, and to the writing of the manuscript. ST, AA, ES, and GD contributed to the design and implementation of the research, and to the review and edits of the manuscript. All authors contributed to the article and approved the submitted version.

## Conflict of Interest

The authors declare that the research was conducted in the absence of any commercial or financial relationships that could be construed as a potential conflict of interest.
