# Supplementary material for: Is COVID-19 Keeping us Up at Night? Stress, Anxiety, and Sleep Among Adult Twins
Source: Front Neurosci. 2021 Apr 26;15:665777. doi: 10.3389/fnins.2021.665777 (PMC8107288; doi:10.3389/fnins.2021.665777)
Supplement: Supplementary file 1 [file Table_1.pdf]

Supplementary Table 1. Select demographic characteristics of individuals in the WSTR who completed a prior registry-wide survey and the current survey.

		Prior survey	Current survey
N		3,797	3,971
Age		49.2 ± 17.2	48.9 ± 17.3
Gender	Men	32%	31%
	Women	68%	69%
Zygosity	MZ	56%	60%
	DZ	44%	50%
Race	White	94	85
	Black	1.8	1.5
	Asian	3	2.7
	American Indian/Alaska Native	1.4	1.6
	Native Hawaiian/Pacific Islander	.6	.6
	Other	1.8	2
Household income[1]	< $20,000	5.2	6.1
	$20,000-$29,999	4.7	4.7
	$30,000-$39,999	5.2	6.1
	$40,000-$49,999	6.1	6.7
	$50,000-$59,999	6.9	6.9
	$60,000-$69,999	6.2	6.2
	$70,000-$79,999	6.6	6.2
	$80,000-$89,999	16	21
	$90,000-$99,999	4.5	3.4
	$100,000-$149,999	14	12
	≥$150,000	17	13

MZ = monozygotic. DZ = dizygotic.

[1] Household income was not assessed in the current survey. The counts and frequencies presented here reflect the individuals' responses to the household income question in their prior survey for those who had completed a prior survey (2,479 individuals who completed the current survey also completed the prior survey).

Note. The prior survey was administered between July 2019 and February 2020; individuals who had been enrolled in the WSTR for at least two years as of July 1 2019 were invited to participate. Response rate was 32.7%. The current survey was administered between March 26 and April 5, 2020. Response rate was 32.6%). Means and standard deviations were presented for age; count and proportions were presented for the categorical variables.

Supplementary Table 2. Model fit differences comparing saturated twin models with nested models for change in sleep amount and sleep quality.

		**Saturated vs. equal thresholds**		**Equal thresholds vs. equal zygosity**	
		Chi square difference	p	Chi square difference	p
Change in sleep amount	No change vs. decrease	2.237	.327	2.605	.457
	No change vs. increase	.445	.801	2.212	.137
Change in sleep quality	No change vs. decrease	.126	.939	0	1.000
	No change vs. increase	.057	.972	1.660	.198

Supplementary Table 3. Model fit differences comparing saturated twin models with nested models for anxiety and perceived stress.

	Saturated vs. equal means		Equal means vs. equal means & variances		Equal means & variances vs. equal zygosity	
	Chi square difference	p	Chi square difference	p	Chi square difference	p
Anxiety	7.10	.029	10.18	.038	21.495	.002
Perceived stress	10.73	.005	11.15	.025	11.54	.073
